# Supplementary material for: Unveiling the Bioactive Potential of the Invasive Jellyfish Phyllorhiza punctata Through Integrative Transcriptomic and Proteomic Analyses
Source: Biomolecules. 2025 Aug 4;15(8):1121. doi: 10.3390/biom15081121 (PMC12383608; doi:10.3390/biom15081121)
Supplement: Supplementary file 1 [file biomolecules-15-01121-s001.zip › Suplementary Material/FileS3/PEPFOLD4/XP_065068843_15126/clusters.html]

 PEP-FOLD clusters


Download archive of all models  here   

|  |  |  |
| --- | --- | --- |
| # | file | sOPEP |
| # cluster 1 - Size: 4 |  |  |
|  | XP\_065068843\_15126-model1 | -37.5558 |
|  | XP\_065068843\_15126-model1.1 | -37.0993 |
|  | XP\_065068843\_15126-model1.2 | -35.0659 |
|  | XP\_065068843\_15126-model1.3 | -26.897 |
| # cluster 2 - Size: 1 |  |  |
|  | XP\_065068843\_15126-model2 | -34.6816 |
| # cluster 3 - Size: 2 |  |  |
|  | XP\_065068843\_15126-model3 | -33.9927 |
|  | XP\_065068843\_15126-model3.1 | -27.7249 |
| # cluster 4 - Size: 5 |  |  |
|  | XP\_065068843\_15126-model4 | -33.6607 |
|  | XP\_065068843\_15126-model4.1 | -31.1488 |
|  | XP\_065068843\_15126-model4.2 | -27.1477 |
|  | XP\_065068843\_15126-model4.3 | -24.9184 |
|  | XP\_065068843\_15126-model4.4 | -23.8297 |
| # cluster 5 - Size: 1 |  |  |
|  | XP\_065068843\_15126-model5 | -32.0002 |
| # cluster 6 - Size: 6 |  |  |
|  | XP\_065068843\_15126-model6 | -30.7251 |
|  | XP\_065068843\_15126-model6.1 | -27.8837 |
|  | XP\_065068843\_15126-model6.2 | -26.9083 |
|  | XP\_065068843\_15126-model6.3 | -25.05 |
|  | XP\_065068843\_15126-model6.4 | -24.1106 |
|  | XP\_065068843\_15126-model6.5 | -23.3916 |
| # cluster 7 - Size: 2 |  |  |
|  | XP\_065068843\_15126-model7 | -30.6137 |
|  | XP\_065068843\_15126-model7.1 | -29.1051 |
| # cluster 8 - Size: 5 |  |  |
|  | XP\_065068843\_15126-model8 | -30.5362 |
|  | XP\_065068843\_15126-model8.1 | -30.0638 |
|  | XP\_065068843\_15126-model8.2 | -29.8777 |
|  | XP\_065068843\_15126-model8.3 | -29.7099 |
|  | XP\_065068843\_15126-model8.4 | -29.1149 |
| # cluster 9 - Size: 2 |  |  |
|  | XP\_065068843\_15126-model9 | -30.2582 |
|  | XP\_065068843\_15126-model9.1 | -29.8501 |
| # cluster 10 - Size: 2 |  |  |
|  | XP\_065068843\_15126-model10 | -30.0256 |
|  | XP\_065068843\_15126-model10.1 | -25.8536 |
| # cluster 11 - Size: 1 |  |  |
|  | XP\_065068843\_15126-model11 | -29.796 |
| # cluster 12 - Size: 2 |  |  |
|  | XP\_065068843\_15126-model12 | -29.505 |
|  | XP\_065068843\_15126-model12.1 | -25.1771 |
| # cluster 13 - Size: 2 |  |  |
|  | XP\_065068843\_15126-model13 | -29.3283 |
|  | XP\_065068843\_15126-model13.1 | -25.9544 |
| # cluster 14 - Size: 1 |  |  |
|  | XP\_065068843\_15126-model14 | -29.0804 |
| # cluster 15 - Size: 12 |  |  |
|  | XP\_065068843\_15126-model15 | -29.041 |
|  | XP\_065068843\_15126-model15.1 | -29.0156 |
|  | XP\_065068843\_15126-model15.2 | -26.4312 |
|  | XP\_065068843\_15126-model15.3 | -26.1015 |
|  | XP\_065068843\_15126-model15.4 | -25.0234 |
|  | XP\_065068843\_15126-model15.5 | -24.9406 |
|  | XP\_065068843\_15126-model15.6 | -24.7622 |
|  | XP\_065068843\_15126-model15.7 | -24.5655 |
|  | XP\_065068843\_15126-model15.8 | -23.6254 |
|  | XP\_065068843\_15126-model15.9 | -23.1355 |
|  | XP\_065068843\_15126-model15.10 | -22.5107 |
|  | XP\_065068843\_15126-model15.11 | -21.4684 |
| # cluster 16 - Size: 2 |  |  |
|  | XP\_065068843\_15126-model16 | -28.7906 |
|  | XP\_065068843\_15126-model16.1 | -26.9642 |
| # cluster 17 - Size: 2 |  |  |
|  | XP\_065068843\_15126-model17 | -28.2662 |
|  | XP\_065068843\_15126-model17.1 | -25.9014 |
| # cluster 18 - Size: 1 |  |  |
|  | XP\_065068843\_15126-model18 | -28.1972 |
| # cluster 19 - Size: 5 |  |  |
|  | XP\_065068843\_15126-model19 | -27.9703 |
|  | XP\_065068843\_15126-model19.1 | -25.6528 |
|  | XP\_065068843\_15126-model19.2 | -23.4917 |
|  | XP\_065068843\_15126-model19.3 | -23.327 |
|  | XP\_065068843\_15126-model19.4 | -21.3005 |
| # cluster 20 - Size: 3 |  |  |
|  | XP\_065068843\_15126-model20 | -27.7777 |
|  | XP\_065068843\_15126-model20.1 | -25.8783 |
|  | XP\_065068843\_15126-model20.2 | -20.8809 |
| # cluster 21 - Size: 1 |  |  |
|  | XP\_065068843\_15126-model21 | -27.2652 |
| # cluster 22 - Size: 2 |  |  |
|  | XP\_065068843\_15126-model22 | -26.7399 |
|  | XP\_065068843\_15126-model22.1 | -24.1613 |
| # cluster 23 - Size: 1 |  |  |
|  | XP\_065068843\_15126-model23 | -26.1793 |
| # cluster 24 - Size: 1 |  |  |
|  | XP\_065068843\_15126-model24 | -26.033 |
| # cluster 25 - Size: 1 |  |  |
|  | XP\_065068843\_15126-model25 | -26.0111 |
| # cluster 26 - Size: 4 |  |  |
|  | XP\_065068843\_15126-model26 | -25.8562 |
|  | XP\_065068843\_15126-model26.1 | -24.6742 |
|  | XP\_065068843\_15126-model26.2 | -21.4415 |
|  | XP\_065068843\_15126-model26.3 | -19.9791 |
| # cluster 27 - Size: 2 |  |  |
|  | XP\_065068843\_15126-model27 | -25.7411 |
|  | XP\_065068843\_15126-model27.1 | -21.1464 |
| # cluster 28 - Size: 2 |  |  |
|  | XP\_065068843\_15126-model28 | -24.4111 |
|  | XP\_065068843\_15126-model28.1 | -22.5562 |
| # cluster 29 - Size: 2 |  |  |
|  | XP\_065068843\_15126-model29 | -24.2418 |
|  | XP\_065068843\_15126-model29.1 | -16.3974 |
| # cluster 30 - Size: 3 |  |  |
|  | XP\_065068843\_15126-model30 | -23.5915 |
|  | XP\_065068843\_15126-model30.1 | -23.2475 |
|  | XP\_065068843\_15126-model30.2 | -20.3617 |
| # cluster 31 - Size: 2 |  |  |
|  | XP\_065068843\_15126-model31 | -23.4449 |
|  | XP\_065068843\_15126-model31.1 | -21.8862 |
| # cluster 32 - Size: 1 |  |  |
|  | XP\_065068843\_15126-model32 | -22.2986 |
| # cluster 33 - Size: 2 |  |  |
|  | XP\_065068843\_15126-model33 | -21.8769 |
|  | XP\_065068843\_15126-model33.1 | -21.8607 |
| # cluster 34 - Size: 3 |  |  |
|  | XP\_065068843\_15126-model34 | -21.4729 |
|  | XP\_065068843\_15126-model34.1 | -21.0956 |
|  | XP\_065068843\_15126-model34.2 | -18.5154 |
| # cluster 35 - Size: 3 |  |  |
|  | XP\_065068843\_15126-model35 | -20.0767 |
|  | XP\_065068843\_15126-model35.1 | -19.0597 |
|  | XP\_065068843\_15126-model35.2 | -18.8083 |
| # cluster 36 - Size: 2 |  |  |
|  | XP\_065068843\_15126-model36 | -19.5006 |
|  | XP\_065068843\_15126-model36.1 | -19.0481 |
| # cluster 37 - Size: 1 |  |  |
|  | XP\_065068843\_15126-model37 | -18.1969 |
| # cluster 38 - Size: 1 |  |  |
|  | XP\_065068843\_15126-model38 | -18.1901 |
| # cluster 39 - Size: 2 |  |  |
|  | XP\_065068843\_15126-model39 | -16.5883 |
|  | XP\_065068843\_15126-model39.1 | -15.8947 |
| # cluster 40 - Size: 1 |  |  |
|  | XP\_065068843\_15126-model40 | -14.7826 |
| # cluster 41 - Size: 1 |  |  |
|  | XP\_065068843\_15126-model41 | -14.5456 |
| # cluster 42 - Size: 1 |  |  |
|  | XP\_065068843\_15126-model42 | -13.0288 |
|  |  |  |
